# Supplementary material for: The Impact of Resident Adipose Tissue Macrophages on Adipocyte Homeostasis and Dedifferentiation
Source: Int J Mol Sci. 2024 Dec 4;25(23):13019. doi: 10.3390/ijms252313019 (PMC11640804; doi:10.3390/ijms252313019)
Supplement: Supplementary file 1 [file ijms-25-13019-s001.zip › Figure S1-S5.pdf]

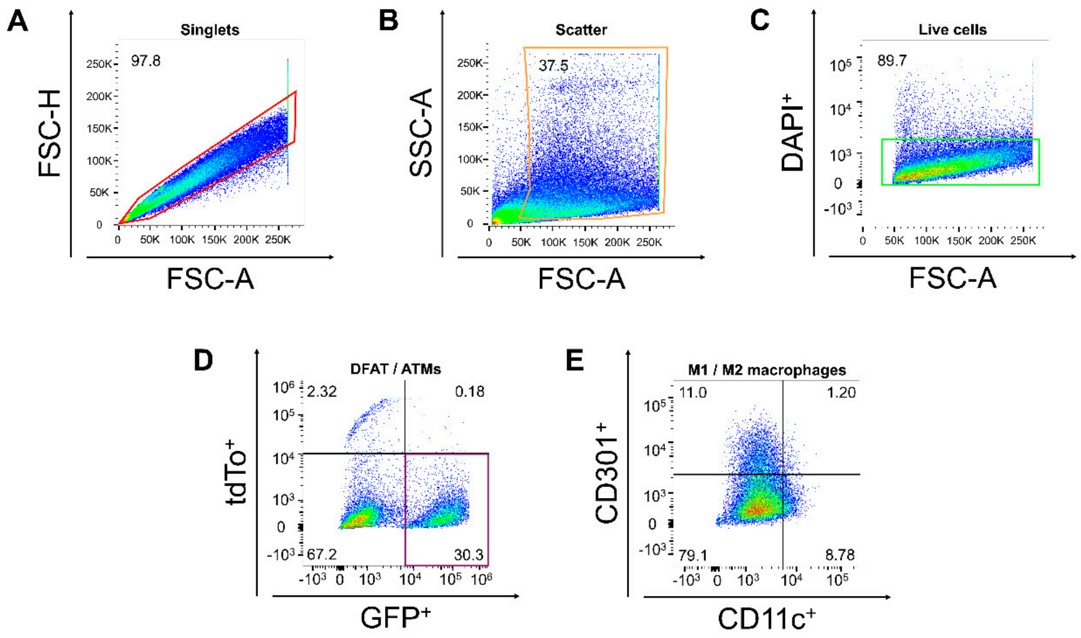

Figure S1: Flow cytometry gating strategy. Representative depiction of the used gating strategy for flow cytometry data analysis. **(A)** Scatter height versus scatter area parameters were used to identify single events. **(B)** Events with a low level of forward scatter (e.g. cell debris, dead cells) were excluded from further analysis. **(C)** 4′,6-Diamidino-2-Phenylindole (DAPI) was used as a viability dye. DAPI-negative events (living stromal vascular fraction (SVF) cells from adipose tissue (AT) explants) were selected for assessment. **(D)** Adipose tissue macrophages (ATMs) and dedifferentiated fat (DFAT) cells were identified gating the green fluorescent protein (GFP) versus tdTomato (tdTo). **(E)** ATM population (GFP^+; $tdTo^-$) was further analysed for macrophage phenotype by gating CD11c (M1) versus CD301 (M2).

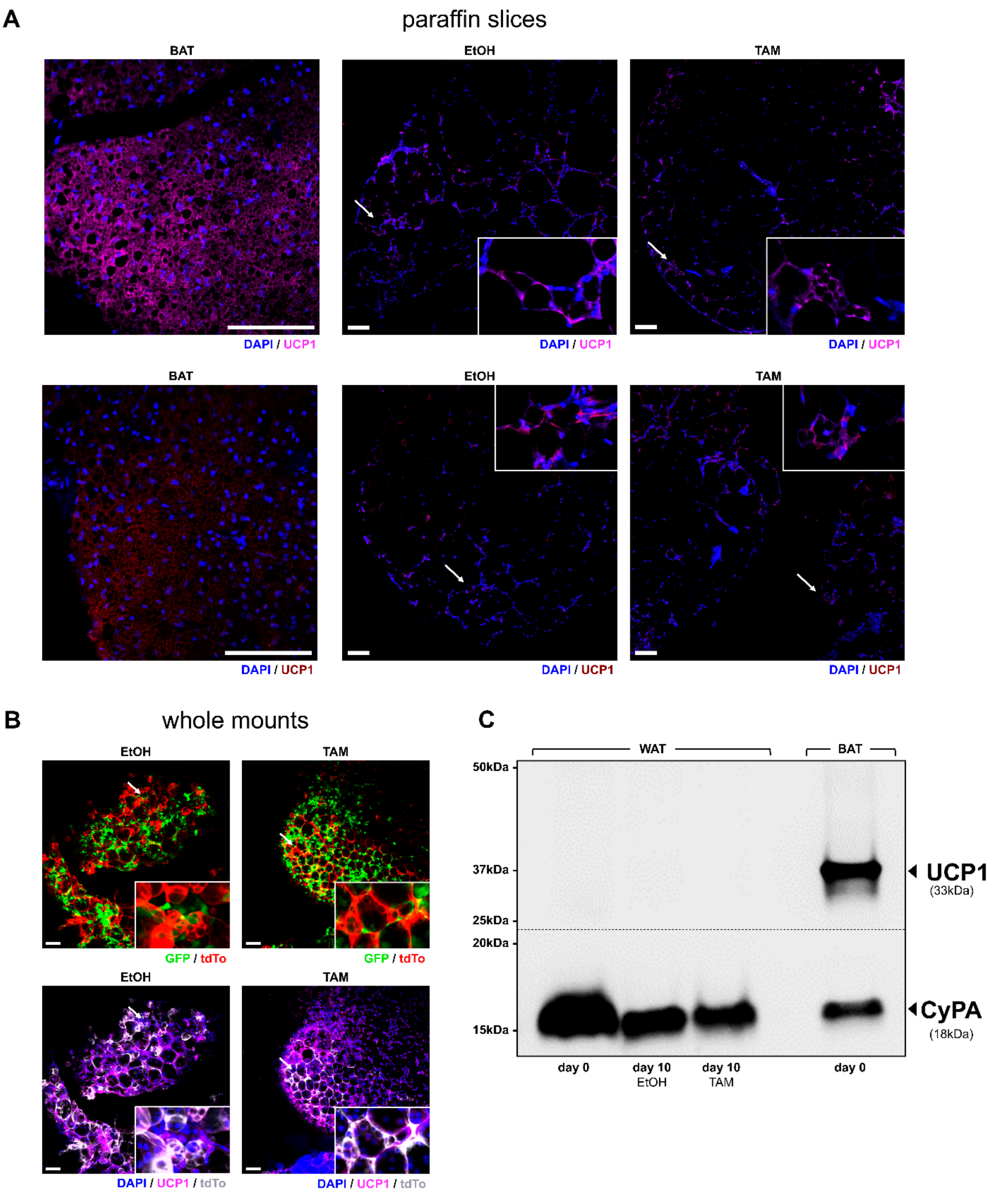

Figure S2: Uncoupling protein 1 (UCP1) detection through immunohistochemical (IHC) staining and western blot. Epididymal white adipose tissue (WAT) explants from homozygous MacFat mice (n = 2-3, N = 1-2) were stimulated with 10 µM tamoxifen (TAM) or 10 µl ethanol (EtOH, control) for 10 days and either prepared for microscopy and IHC staining (paraffin embedding or whole mounts) or processed for western blot analysis. **(A)** Microscopy imaging of TAM stimulated and non-stimulated (EtOH) AT explant paraffin sections (blue: DAPI, purple/red: UCP1) showed no increase in UCP1 expression through TAM stimulation (n = 3, N = 1). Upper row used an AF647-conjugated secondary antibody, lower row an Cy3-conjugated secondary antibody. Brown adipose tissue (BAT, left) was used as positive control. **(B)** Microscopy imaging of TAM stimulated and non-stimulated (EtOH) AT explant whole mounts (red/grey: adipocytes, green: ATMs, blue: DAPI, purple: UCP1) likewise showed no UCP1 increase under TAM (n = 3, N = 2). **(C)** UCP1 was not detectable in epididymal WAT explants from homozygous MacFat mice through western blot no matter the stimulation condition (n = 2, N = 1). BAT from the same genotype showed clear UCP1 expression. Densitometry was performed to compare expression levels. CyPA was used as loading control. Scale bars = 100 µm.

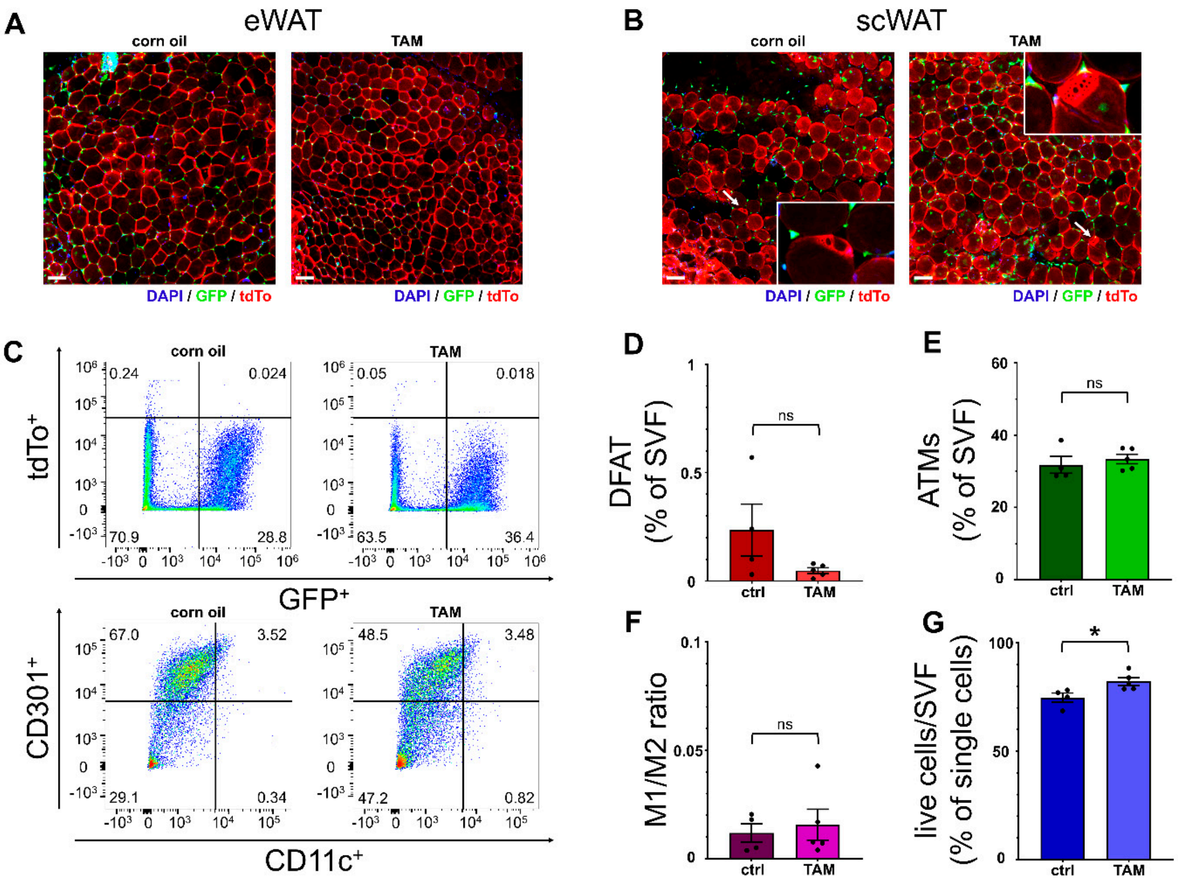

Figure S3: In vivo tamoxifen (TAM) stimulation of homozygous MacFat mice. Homozygous MacFat mice were intraperitoneally injected for 5 days with 100 µl corn oil (control) or 1 mg TAM per day (5mg in total) (n = 5 per condition, N = 2) and sacrificed on day 6. **(A, B)** Microscopy imaging of TAM stimulated and non-stimulated (A) epididymal white adipose tissue (eWAT) and (B) subcutaneous white adipose tissue (scWAT) whole mounts (red: adipocytes, green: ATMs) showed no difference in DFAT cell appearance. **(C)** Representative flow cytometry dot plots showing DFAT cells (GFP^-; $tdTo^+$;) and ATMs (GFP^+; $tdTo^-$) (upper row) as well as macrophage phenotype (M1: $CD11c^+$; $CD301^-$ / M2: $CD11c^-$; $CD301^+$) (lower row) after TAM injection. **(D)** Proportion of DFAT cells (GFP^-; $tdTo^+$) from SVF. **(E)** Proportion of ATMs (GFP^+; $tdTo^-$) from SVF. **(F)** Ratio of pro- (M1) to anti-inflammatory (M2) macrophages. **(G)** Proportion of live cells (DAPI-negative) from counted single cells increased with TAM injection. Data represented as mean ±SEM. Scale bars = 100 µm. * p-value < 0.05

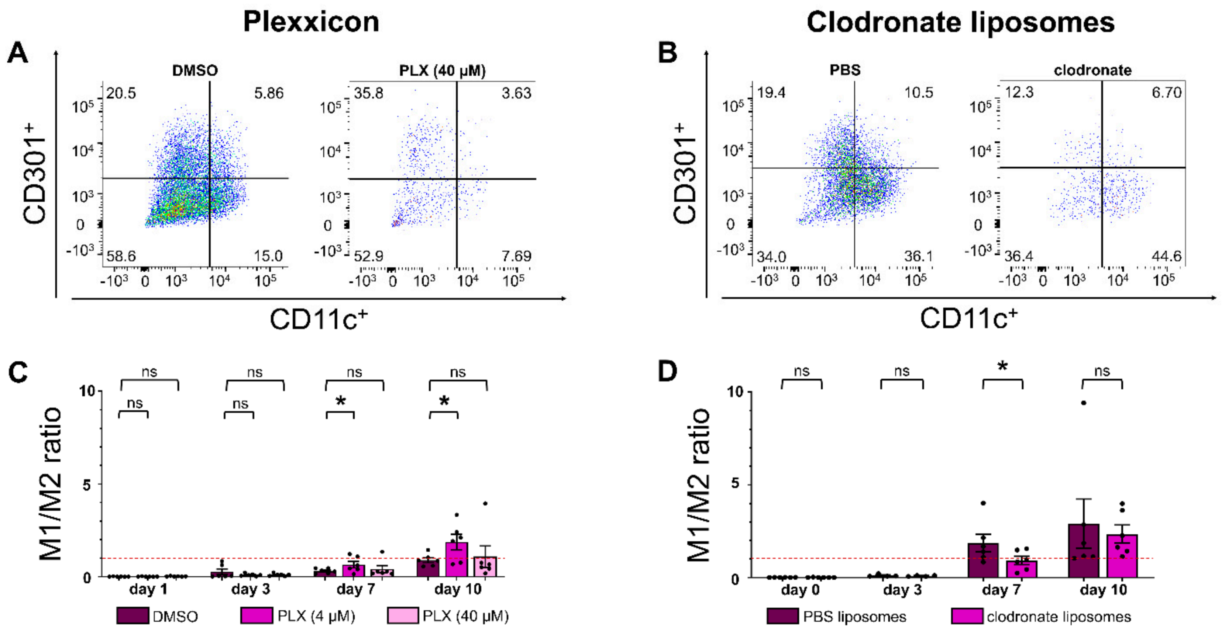

Figure S4: Ratio of pro- to anti-inflammatory ATMs under macrophage depleting conditions. Epididymal WAT explants from homozygous MacFat mice (n = 6 , N = 3 per condition) were cultivated under ATM depleting conditions through Plexxicon 5622 (PLX) or clodronate liposomes for 10 days. **(A, B)** Representative flow cytometry dot plots showing overall macrophage depletion as well as macrophage phenotype distribution (M1: $CD11c^{+}$; $CD301^{-}$ / M2: $CD11c^{-}$; $CD301^{+}$) on day 10 after (A) PLX or (B) clodronate liposome stimulation. **(C, D)** Ratio of pro- (M1) to anti-inflammatory (M2) macrophages on different timepoints during cultivation showed a pro-inflammatory shift after (C) PLX stimulation and an anti-inflammatory shift after (D) clodronate liposome stimulation. Red line marks a balanced 1:1 ratio. Data represented as mean ±SEM. * p-value < 0.05

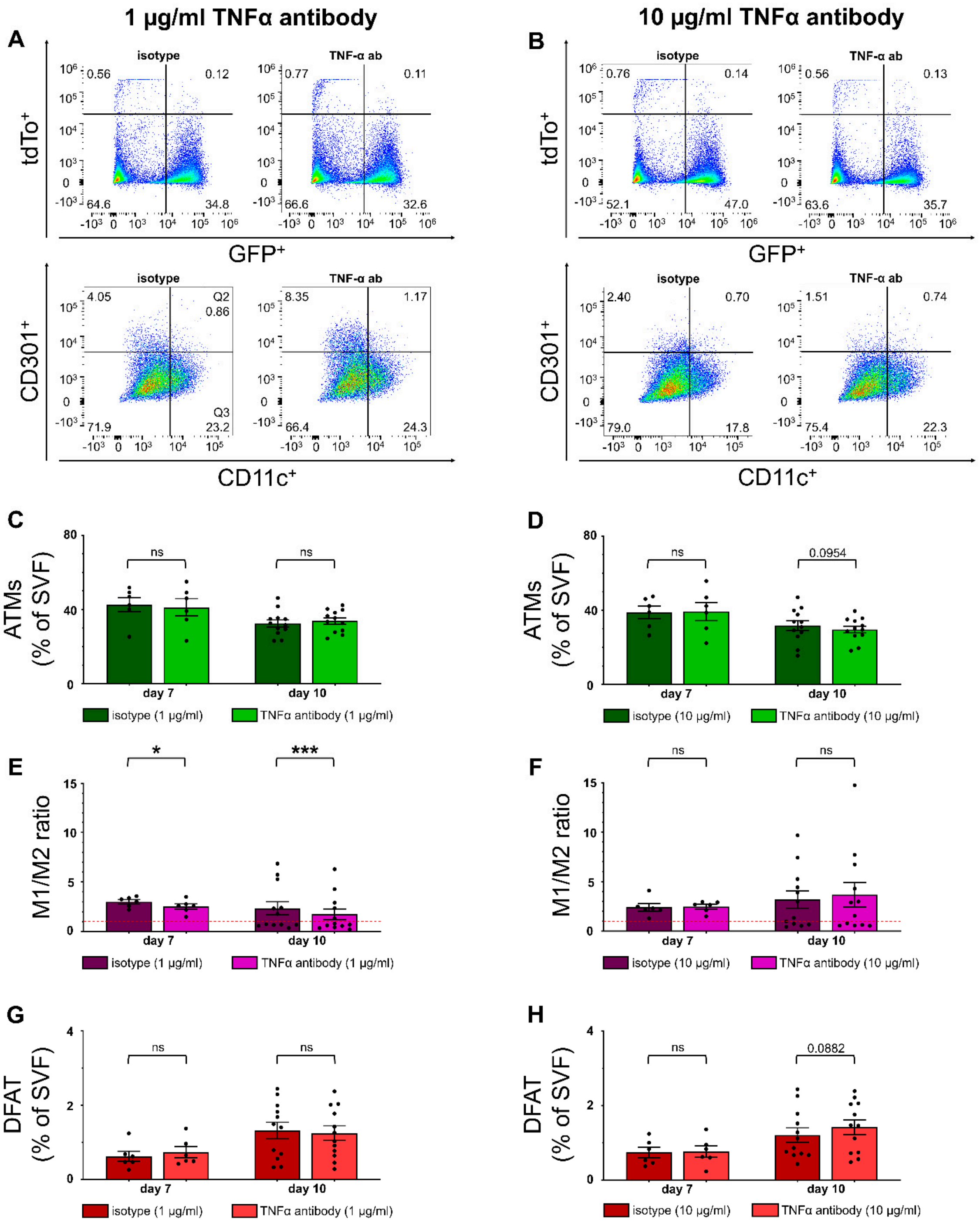

Figure S5: Application of Tumor necrosis factor α (TNFα) neutralizing antibodies shows a trend of DFAT cell increase in explant culture. TNFα neutralizing antibodies were applied in explant culture of epididymal WAT explants from homozygous MacFat mice (n = 6, N = 3 for day 7; n = 12, N = 6 for day 10) in the concentrations 1 or 10 µg/ml. Normal goat IgG was used as isotype control. Results from 1 µg/ml antibody are shown on the left, results from 10 µg/ml antibody on the right. **(A, B)** Representative flow cytometry dot plots showing DFAT cells (GFP$^-$; tdTo$^+$) and ATMs (GFP$^+$; tdTo$^-$) (upper rows) as well as the ratio of pro- (CD11c$^+$; CD301$^-$) to anti-inflammatory (CD11c$^-$; CD301$^+$) macrophages (lower rows) after application of (A) 1 µg/ml or (B) 10 µg/ml TNFα antibody. **(C, D)** Proportion of ATMs (GFP$^+$; tdTo$^-$) from SVF on different time points during cultivation after application of (C) 1 µg/ml or (D) 10 µg/ml TNFα antibody. **(E, F)** Ratio of pro- (M1) to anti-inflammatory (M2) macrophages on different time points during cultivation after application of (E) 1 µg/ml or (F) 10 µg/ml TNFα antibody. Red line marks a balanced 1:1 ratio. **(G, H)** Proportion of DFAT cells (GFP$^-$; tdTo$^+$) from SVF on different time points during cultivation after application of (G) 1 µg/ml or (H) 10 µg/ml TNFα antibody. Data shown as mean ±SEM. Scale bars = 100µm. * p-value < 0.05; *** p-value < 0.001.
