# Supplementary material for: The Impact of Resident Adipose Tissue Macrophages on Adipocyte Homeostasis and Dedifferentiation
Source: Int J Mol Sci. 2024 Dec 4;25(23):13019. doi: 10.3390/ijms252313019 (PMC11640804; doi:10.3390/ijms252313019)
Supplement: Supplementary file 1 [file ijms-25-13019-s001.zip › S7 - The ARRIVE Essential 10 - ijms submission 3312254.pdf]

The ARRIVE Essential 10

These items are the basic minimum to include in a manuscript. Without this information, readers and reviewers cannot assess the reliability of the findings.

Item		Recommendation	Section/line number, or the reasoning for not reporting
**Study design**	1	For each experiment, provide brief details of study design including:	
		a. The groups being compared, including control groups. If no control group has been used, the rationale should be stated.	Control groups are stated in the respective figure texts.
		b. The experimental unit (e.g. a single animal, litter, or cage of animals).	Mice functioned as experimental units. Concrete animal numbers are provided in the respective figure texts.
**Sample size**	2	a. Specify the exact number of experimental units allocated to each group, and the total number in each experiment. Also indicate the total number of animals used.	Concrete animal numbers are provided in the respective figure texts. In total, 68 mice were used.
		b. Explain how the sample size was decided. Provide details of any a priori sample size calculation, if done.	Sample size was based on previous experiments.
**Inclusion and exclusion criteria**	3	a. Describe any criteria used for including and excluding animals (or experimental units) during the experiment, and data points during the analysis. Specify if these criteria were established a priori. If no criteria were set, state this explicitly.	Animals were chosen for the experiments based on sex, age and genotype. Data points marked as outliers in statistical analysis (ROUT, Q = 1%) were excluded.
		b. For each experimental group, report any animals, experimental units or data points not included in the analysis and explain why. If there were no exclusions, state so.	All data points not marked as outliers were included.
		c. For each analysis, report the exact value of n in each experimental group.	Concrete animal numbers per group are provided in the respective figure texts.
**Randomisation**	4	a. State whether randomisation was used to allocate experimental units to control and treatment groups. If done, provide the method used to generate the randomisation sequence.	No randomisation was used.
		b. Describe the strategy used to minimise potential confounders such as the order of treatments and measurements, or animal/cage location. If confounders were not controlled, state this explicitly.	Since there were no obvious confounders, confounders were not controlled.
**Blinding**	5	Describe who was aware of the group allocation at the different stages of the experiment (during the allocation, the conduct of the experiment, the outcome assessment, and the data analysis).	No additional blinding of the respective investigators was performed.
**Outcome measures**	6	a. Clearly define all outcome measures assessed (e.g. cell death, molecular markers, or behavioural changes).	Number of live cells, dedifferentiated adipocytes, adipose tissue macrophages and macrophage phenotype was assed.
		b. For hypothesis-testing studies, specify the primary outcome measure, i.e. the outcome measure that was used to determine the sample size.	Number of dedifferentiated adipocytes was used to estimate sample size based on previous experiments.
**Statistical methods**	7	a. Provide details of the statistical methods used for each analysis, including software used.	Statistical methods are described in the under point 4.11 (line 694-704) of the materials and methods section.
		b. Describe any methods used to assess whether the data met the assumptions of the statistical approach, and what was done if the assumptions were not met.	Data were tested for Gaussian distribution by the Shapiro-Wilk test to meet requirements for further statistical evaluation.

**Experimental animals**	8	a. Provide species-appropriate details of the animals used, including species, strain and substrain, sex, age or developmental stage, and, if relevant, weight.	Animal details are provided under point 4.1 (line 526-541) of the materials and methods section.
		b. Provide further relevant information on the provenance of animals, health/immune status, genetic modification status, genotype, and any previous procedures.	Animal details are provided under point 4.1 (line 526-541) of the materials and methods section.
**Experimental procedures**	9	For each experimental group, including controls, describe the procedures in enough detail to allow others to replicate them, including:	
		a. What was done, how it was done and what was used.	Details provided in the manuscript.
		b. When and how often.	Details provided in the manuscript.
		c. Where (including detail of any acclimatisation periods).	Details provided in the manuscript.
		d. Why (provide rationale for procedures).	Details provided in the manuscript.
**Results**	10	For each experiment conducted, including independent replications, report:	
		a. Summary/descriptive statistics for each experimental group, with a measure of variability where applicable (e.g. mean and SD, or median and range).	Details provided in the manuscript. SEM was used as measure of variability.
		b. If applicable, the effect size with a confidence interval.	Not applicable.
